# Early Patient‐Reported Outcomes as Predictors of Long‐Term Scar Satisfaction: An Exploratory Cohort Study

**DOI:** 10.1111/wrr.70094

**Published:** 2025-09-23

**Authors:** Antoinette Nguyen, Rena Li, Robert Galiano

**Affiliations:** ^1^ University of Rochester School of Medicine Rochester New York USA; ^2^ Northwestern University Feinberg School of Medicine Chicago Illinois USA

## Abstract

Early prediction of long‐term scar outcomes is essential for guiding clinical decision‐making and improving patient satisfaction. This study investigates whether 3‐month patient‐reported outcomes (PROs) using SCAR‐Q domains—psychosocial, appearance and quality of life (QoL)—predict 12‐month outcomes, and how these relate to objective scar measures. A prospective cohort of 20 female patients undergoing various surgical procedures completed SCAR‐Q and Patient and Observer Scar Assessment Scale (POSAS) evaluations at 3 and 12 months postoperatively. Correlation and linear regression analyses assessed associations and predictive validity between early and late scar outcomes. SCAR‐Q QoL scores demonstrated strong predictive validity (*R* ^2^ = 0.49, *p* < 0.001; *ρ* = 0.70, *p* < 0.001), whereas psychosocial and appearance domains showed weak, nonsignificant associations (*R* ^2^ = 0.12 and 0.10, respectively; *p* > 0.1). Objective scar characteristics—particularly width and height—were significantly correlated with poorer 12‐month appearance and psychosocial scores (e.g., *ρ* = −0.743 for height vs. appearance, *p* < 0.001; *ρ* = −0.605 for width vs. psychosocial, *p* = 0.0047). In point‐biserial correlations, wider and taller scars at 3 months were more likely to be rated as ‘bad’ at 12 months (*r* ≥ |0.53|, *p* ≤ 0.016). POSAS and overall opinion scores also significantly improved over time (*p* < 0.05), but some patients reported increased appearance‐related distress despite objective improvements. In conclusion, early QoL assessments reliably predict long‐term outcomes, while appearance and psychosocial perceptions may shift over time. These findings support routine use of PROs in early postoperative care to inform personalised interventions and optimise scar management.

## 1 Introduction

Scarring represents an intrinsic aspect of the wound‐healing process, involving a complex interplay of biological, physiological and environmental determinants [1, 2, 3]. The wound‐healing process is typically delineated into three primary phases: inflammation, proliferation and remodelling. Each of these phases significantly contributes to scar characteristics, including texture, colour and overall appearance [4, 5, 6]. The inflammatory phase, which commences immediately following injury, involves the recruitment of immune cells that facilitate debris clearance and infection prevention [7]. The subsequent proliferation phase is characterised by the formation of granulation tissue, collagen synthesis and wound contraction [8]. Finally, the remodelling phase involves the reorganisation and maturation of collagen fibres, resulting in progressive changes to scar texture and elasticity [9]. Environmental influences, such as ultraviolet (UV) radiation exposure and skincare practices, alongside genetic predisposition, also critically shape scar characteristics [10, 11, 12]. These complex, multifactorial dynamics ultimately impact patient quality of life, underscoring the importance of effective scar management in post‐injury care.

Historically, clinical scar assessment has predominantly relied on objective measures such as scar height, vascularity and pliability, in conjunction with subjective clinician evaluations. Although these methods provide critical insights into the physical attributes of scars, they often fail to capture the comprehensive spectrum of patient experiences and satisfaction with their scars. Patient‐reported outcomes (PROs) have therefore emerged as crucial indicators of treatment efficacy, offering nuanced insights into patients' perceptions of their scars and overall satisfaction. Instruments such as the Scar‐Q and the Patient and Observer Scar Assessment Scale (POSAS) have been developed to evaluate both the physical and psychosocial dimensions of scarring [13, 14, 15]. By integrating PROs into scar management protocols, healthcare providers can better understand patients' needs and tailor interventions more effectively, thereby enhancing the quality of care.

Despite the increasing recognition of PROs in clinical practice, there remains a considerable gap in the literature concerning the predictive value of early PRO assessments [16]. Specifically, the potential of PROs collected at 3 months post‐injury to predict long‐term satisfaction and outcomes at 12 months has yet to be comprehensively investigated. Establishing this predictive relationship could significantly enhance clinical decision‐making, enabling early interventions for patients at risk of long‐term dissatisfaction. Moreover, early identification of potential issues could facilitate timely psychological support, proactive scar treatment and other targeted interventions, ultimately improving long‐term patient outcomes [17, 18].

The objective of this study is to determine whether PROs, as measured by the SCAR‐Q and POSAS at 3 months, can reliably predict long‐term scar satisfaction and outcomes at 12 months. This exploratory study intentionally included a heterogeneous variety of surgical scars to broadly assess how early patient‐reported outcomes may forecast long‐term perceptions across different healing contexts. Establishing such predictive relationships would have substantial implications for patient‐centred care, enabling clinicians to formulate personalised care plans based on early patient feedback. Ultimately, this approach could mitigate dissatisfaction, enhance mental health outcomes and optimise overall patient quality of life. By addressing these gaps, this study seeks to contribute valuable insights into scar management practices, ensuring that both the physical and psychological aspects of healing are adequately addressed.

## 2 Methods

This study was a prospective observational cohort study conducted to evaluate the predictive value of early PROs in scar management. Twenty female participants were recruited based on specific inclusion criteria, focusing on patients aged 18 and over. Exclusion criteria included: pre‐existing skin conditions (e.g., chronic dermatitis), systemic inflammatory or autoimmune disorders, uncontrolled psychiatric conditions, prior reoperations at the scar site, inability to provide informed consent, receipt of scar‐modifying therapeutics (such as corticosteroid injections or laser therapy) and lack of follow‐up data at the 12‐month mark. Twelve‐month outcomes were selected because scars typically reach maturation by this time, and substantial changes in appearance or quality are unlikely to occur beyond 1 year postoperatively. A power analysis was performed prior to recruitment, indicating that a sample size of 20 participants would provide sufficient power to detect moderate effect sizes in correlation and regression analyses.

PROs were assessed at two different time points: 3 months and 12 months post‐injury. The SCAR‐Q, a rigorously validated, patient‐reported outcome instrument, was used to assess multiple dimensions of scarring, including psychosocial burden, appearance‐related distress and scar‐specific quality of life. This tool is designed specifically for patients with surgical or traumatic scars and captures subjective experiences across three core domains. The psychosocial subscale measures the emotional and social impact of scarring (e.g., embarrassment, self‐consciousness), while the appearance subscale evaluates dissatisfaction or distress related to how the scar looks. The QoL subscale assesses how the scar interferes with aspects of daily life, such as clothing choices or physical activities. Scores were calculated using the Rasch‐transformed SCAR‐Q scoring system, with higher values indicating better perceived outcomes. These PROs were collected prospectively and paired with corresponding objective scar assessments to allow for temporal and cross‐domain comparisons [19]. Additionally, the POSAS was employed to evaluate the quality of scars based on patient perceptions and clinical assessments, encompassing domains such as scar colour, thickness and relief. Patients completed the Scar‐Q and POSAS questionnaires during follow‐up visits at both 3‐month and 12‐month intervals [20]. Objective scar measures, including vascularity, height, width and depth, were recorded during clinical evaluations at both time points.

Objective scar assessments were conducted by two board‐certified plastic surgeons using standardised measurement techniques. Vascularity was scored on a scale of 0 (normal) to 4 (very red), height and width were measured in millimetres and depth was assessed using a standardised device. To assess inter‐rater reliability, intraclass correlation coefficients (ICC) were computed for vascularity, width, height and depth using a two‐way random‐effects model with absolute agreement (ICC[A, 2]). ICC values exceeded 0.4, indicating at least moderate agreement between raters. Descriptive statistics were calculated for all variables at both time points to summarise the data. The relationship between early PRO scores at 3 months and long‐term satisfaction and outcomes at 12 months was assessed using linear regression and Pearson or Spearman correlation coefficients, depending on the normality of the data. Paired *t*‐tests were employed to analyse differences in scores between the two time points. A *p*‐value of < 0.05 was considered statistically significant for all analyses, which were performed using R (version 4.4.2).

## 3 Results

### 3.1 Demographics

Among the 20 participants in this study, six identified as African‐American and 14 as Caucasian. All participants were female (Table 1). Fourteen participants underwent breast‐related surgeries, including eight who had bilateral breast reductions. Other procedures involved were brachioplasty, abdominoplasty, Mohs closure, lipoma removal, breast revision, implant exchange and breast wound debridement. A broad range of surgical procedures was purposefully included to enhance the generalisability of scar‐related findings across different types of surgical wounds and healing contexts.

**TABLE 1:** Patient demographics and baseline characteristics.

Demographics	Participants (*N*)	%
Race		
Caucasian	13	65%
African‐American	6	30%
Hispanic	1	5%
Age		
18–40	9	45%
41–65	7	35%
65+	4	20%

### 3.2 Descriptive Statistics

**FIGURE 1:**
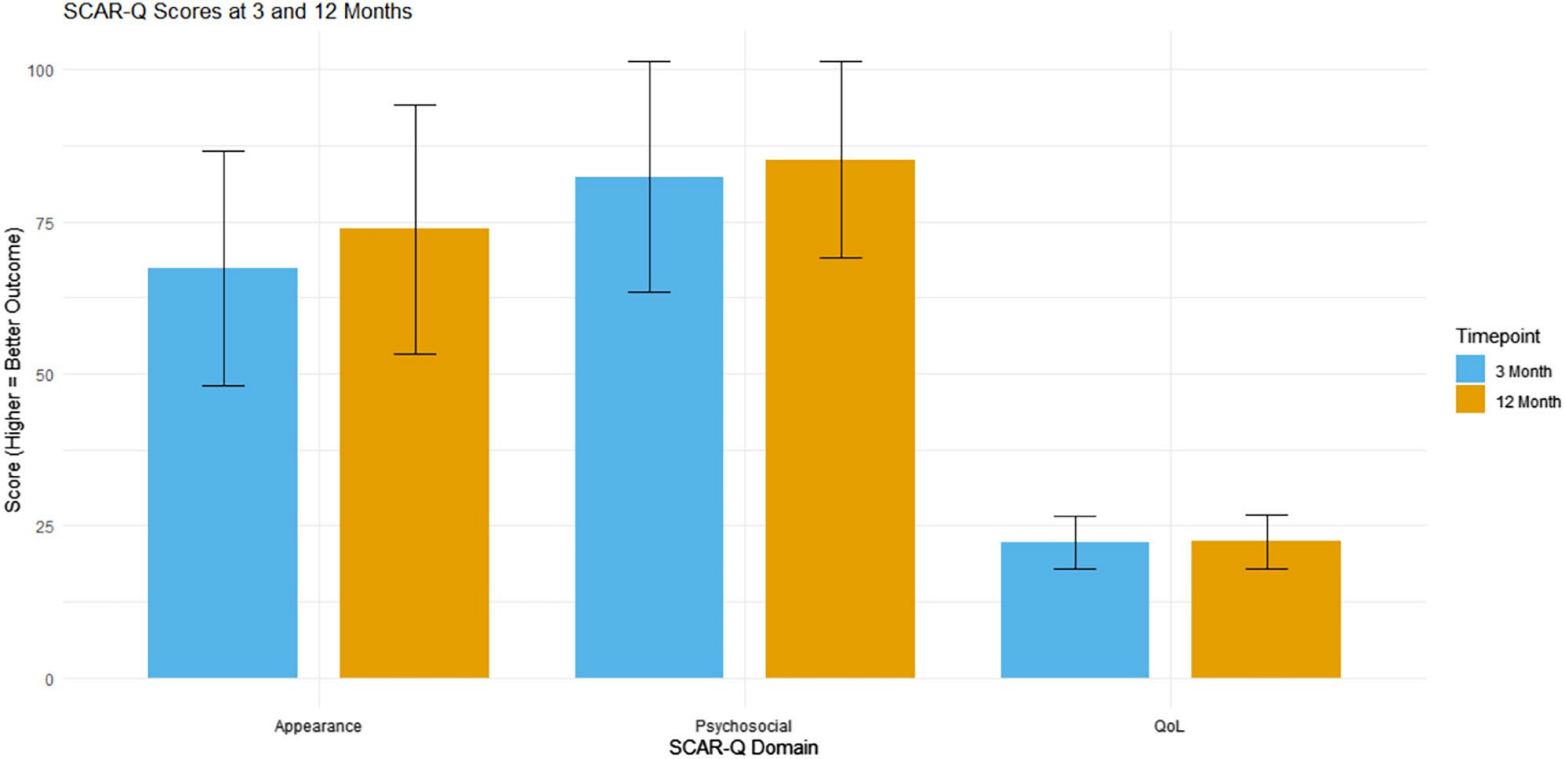
Bar plot comparing patient‐reported SCAR‐Q outcomes at 3 and 12 months postoperatively across three domains: psychosocial impact, appearance and quality of life (QoL). Higher scores represent more favourable outcomes. Error bars represent standard deviations.

For the SCAR‐Q measures, the psychosocial score (ranging 0–100, with higher scores indicating better psychosocial outcomes) increased from a mean of 82.35 at 3 months to 85.20 at 12 months (SD = 18.90 at 3 months, 16.05 at 12 months; mean difference 2.85, *t*(19) = −0.616, *p* = 0.5452). The appearance score (also 0–100, with higher values reflecting more favourable appearance) rose from 67.25 at 3 months to 73.80 at 12 months (mean difference 6.55, *t*(19) = −1.219, *p* = 0.2377). Meanwhile, quality of life (QoL), measured on a 0–28 scale (with 28 denoting highest QoL), shifted from 22.25 ± 4.4 at 3 months to 22.35 ± 4.4 at 12 months—indicating an extremely small and not statistically significant improvement (mean difference 0.10, *t*(19) = −0.129, *p* = 0.899) (Figure 1).

**FIGURE 2:**
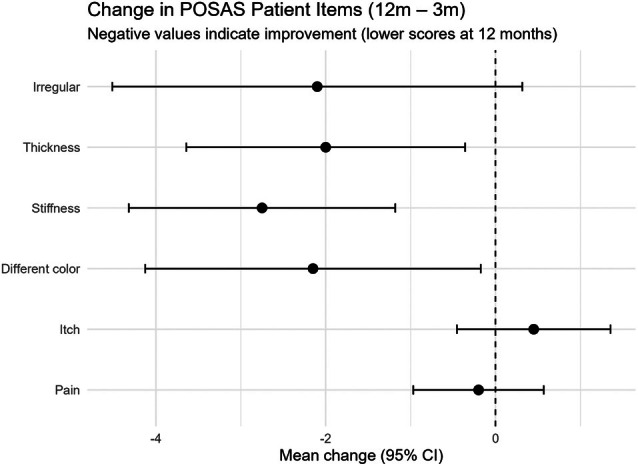
Change in POSAS patient‐scale items from 3 to 12 months postoperatively. Mean change (12‐month score minus 3‐month score) is shown for each POSAS patient‐scale item, with error bars representing 95% confidence intervals. Negative values indicate improvement (i.e., lower scores at 12 months, reflecting better scar quality in the POSAS scoring convention).

For the POSAS patient scale, the sum score significantly decreased from 3 to 12 months (mean change = −8.75, *p* = 0.0269), indicating an overall improvement in scar quality over time. Item‐level analysis showed that the largest mean decreases were in stiffness (−2.75, *p* = 0.0016), different colour (−2.15, *p* = 0.0345) and thickness (−2.00, *p* = 0.0196), suggesting these parameters contributed most to the overall improvement (Figure 2). Likewise, patients' overall opinion of scar quality (assessed on a 0–10 scale, where 10 indicates a severe difference from normal skin) decreased from 4.55 at 3 months to 3.85 at 12 months (*p* = 0.0194), suggesting participants perceived noticeably better scar outcomes over time.

### 3.3 Correlation Analysis

**FIGURE 3:**
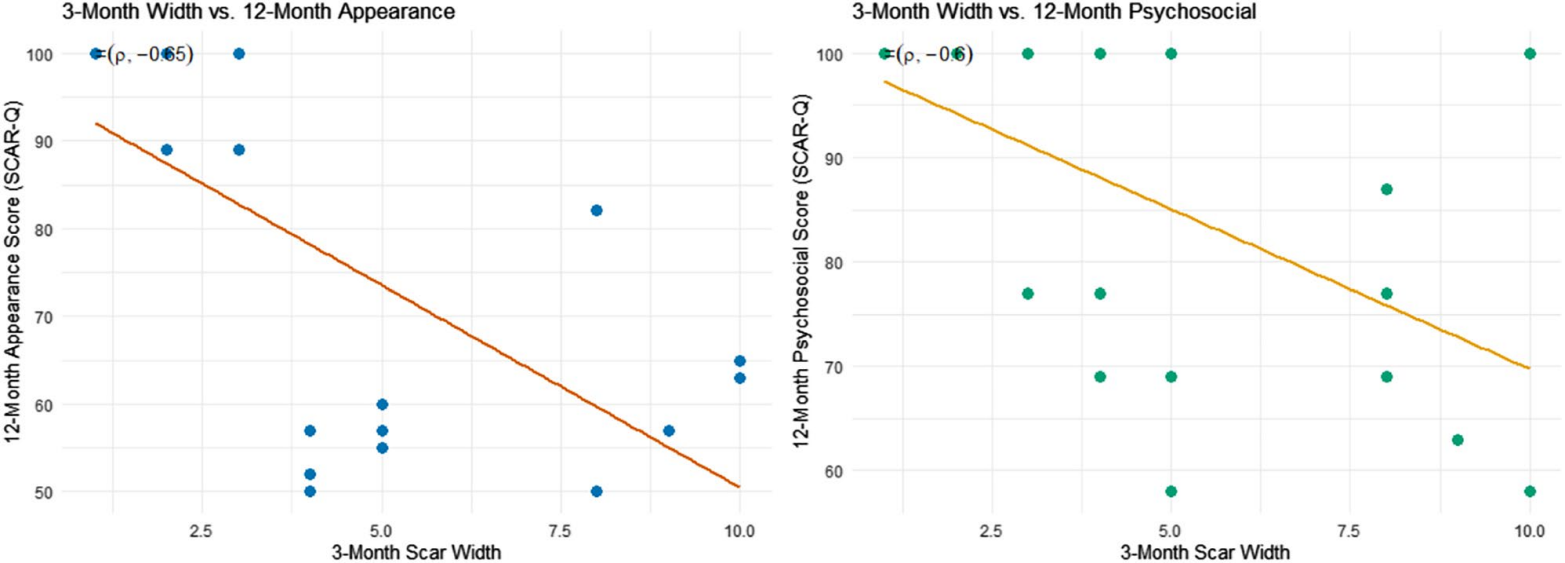
Associations between early scar width and patient‐reported outcomes at 12 months. Scatterplots demonstrate the relationship between 3‐month scar width and two domains of the SCAR‐Q at 12 months: (A) Appearance scores and (B) Psychosocial scores. Wider scars at 3 months were significantly associated with lower patient‐reported appearance scores (Spearman's *ρ* = −0.647, *p* = 0.002) and lower psychosocial well‐being (Spearman's *ρ* = −0.605, *p* = 0.0047) at 12 months. Each point represents a single patient. Higher SCAR‐Q scores indicate more favourable patient‐reported outcomes.

A series of correlation analyses were performed to examine how early scar metrics related to patient‐reported outcomes at both 3 and 12 months, with higher SCAR‐Q scores indicating more favourable psychosocial, quality‐of‐life or appearance perceptions. Overall, scar width and height at 3 months demonstrated moderate to strong associations with 12‐month patient‐reported appearance and psychosocial outcomes, whereas depth and vascularity exhibited minimal links to SCAR‐Q scores at either time point. Notably, 3‐month width negatively correlated with both 12‐month appearance (*ρ* = −0.647, *p* = 0.002) and psychosocial (*ρ* = −0.605, *p* = 0.0047) scores, suggesting that wider scars early in healing tend to receive lower patient‐reported ratings at 12 months (Figure 3). Similarly, 3‐month height correlated strongly and negatively with 12‐month appearance (*ρ* = −0.743, *p* < 0.001), while the point‐biserial correlation showed taller 3‐month scars more frequently being classified as ‘bad’ (*r* = −0.530, *p* = 0.016). In contrast, depth remained stable between 3 and 12 months (*ρ* = 1.00, *p* < 0.001), implying little change in scar depth over time and vascularity rarely reached statistical significance, indicating limited impact on long‐term patient satisfaction for this cohort.

**FIGURE 4:**
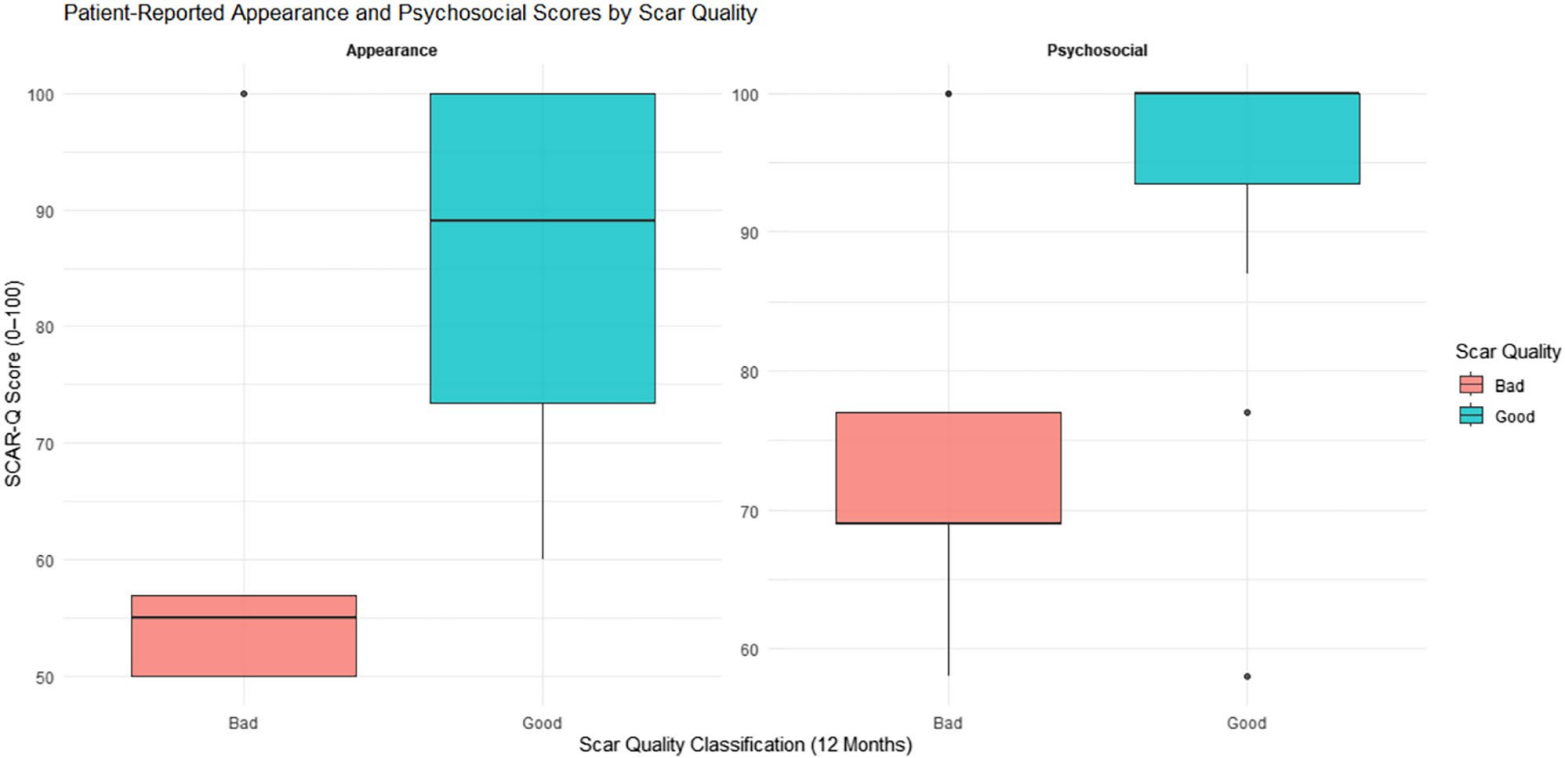
Boxplots of patient‐reported SCAR‐Q appearance and psychosocial scores at 12 months. They are stratified by binary scar quality classification (‘good’ vs. ‘bad’). Patients who were classified as having ‘good’ scars reported significantly higher appearance (*r* = 0.67, *p* = 0.0013) and psychosocial scores (*r* = 0.53, *p* = 0.016), indicating better aesthetic and emotional outcomes. Higher SCAR‐Q scores reflect better patient‐reported quality.

Within single time points, width consistently showed an inverse relationship with patient‐reported appearance or psychosocial well‐being—most strongly at 12 months (e.g., *ρ* = −0.693, *p* < 0.001 with appearance). Appearance and psychosocial scores themselves were positively correlated at both intervals (e.g., *ρ* = 0.812, *p* < 0.0001 at 3 months), reinforcing that when patients perceive their scar's appearance as better, they also tend to report higher psychosocial satisfaction. Although many correlations involving depth or vascularity were nonsignificant, the negative association between 3‐month vascularity and 12‐month height (*ρ* = −0.449, *p* = 0.047) suggests that some vascular abnormalities early on may align with less scar height at 12 months. Additionally, several 3‐month metrics (e.g., height, width) were strongly predictive of being rated ‘bad’ at 12 months, whereas high appearance or psychosocial scores often tracked with a ‘good’ classification (*r* ≥ |0.53|, *p* ≤ 0.016) (Figure 4). In contrast, some pairs (e.g., 3‐month quality of life vs. 12‐month width, 3‐month depth vs. 12‐month appearance) demonstrated no meaningful associations. Collectively, these findings indicate that, among the SCAR‐Q patient‐reported domains, appearance and psychosocial dimensions strongly correlate with certain early objective scar measures—particularly width and height—while depth and vascularity appear less influential in shaping long‐term patient satisfaction.

#### 3.3.1 Regression Analysis

**FIGURE 5:**
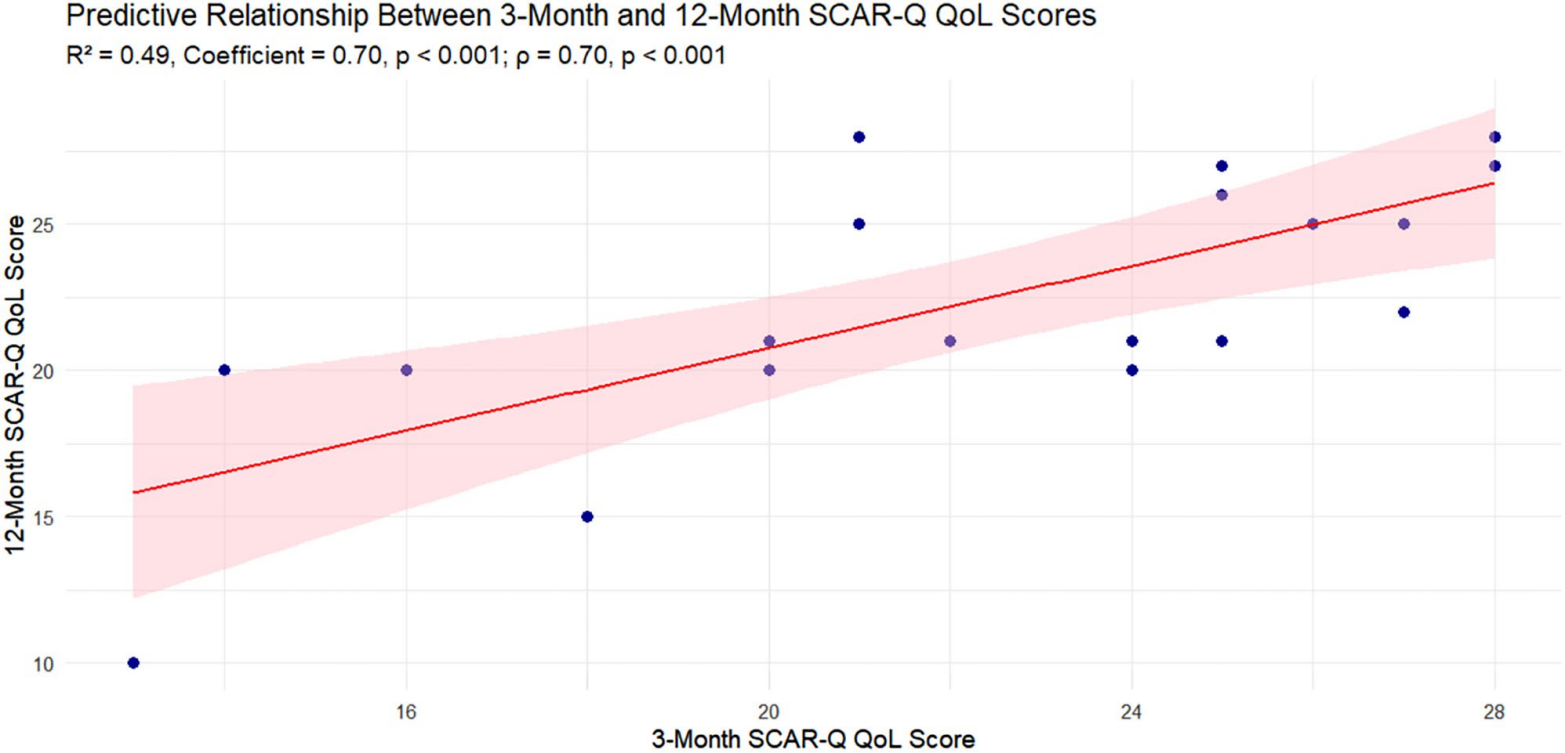
Predictive relationship between 3‐month and 12‐month SCAR‐Q Quality of Life (QoL) scores: This scatterplot illustrates the linear association between patient‐reported QoL scores at 3 months and 12 months postoperatively. Each point represents an individual patient. The red line represents the linear regression fit with a shaded 95% confidence interval. The model demonstrated strong predictive validity, with 3‐month QoL scores significantly predicting 12‐month outcomes (*R* ^2^ = 0.49, *p* < 0.001). A Spearman correlation (*ρ* = 0.70, *p* < 0.001) confirmed a robust positive relationship between early and long‐term QoL assessments.

Regression and correlation analyses were conducted to evaluate the predictive validity of 3‐month SCAR‐Q patient‐reported outcomes—psychosocial impact, appearance‐related distress and quality of life (QoL)—on corresponding 12‐month scores. SCAR‐Q quality of life (QoL) scores showed strong predictive validity. The linear regression model demonstrated that 3‐month QoL scores explained nearly half of the variance in 12‐month QoL (*R* ^2^ = 0.49), with a significant coefficient of 0.70 (*p* < 0.001). Spearman correlation corroborated this finding, with a robust positive association (*ρ* = 0.70, *p* < 0.001) (Figure 5). This suggests that patients' perceived quality of life related to their scars is relatively stable over time and can be accurately forecasted based on early postoperative assessments. Additionally, the overall opinion of scar quality demonstrated a strong regression model (*R* ^2^ = 0.527) with a coefficient estimate of 1.036 and a highly significant *p*‐value (*p* < 0.0003).

Conversely, for SCAR‐Q psychosocial scores, the linear regression model demonstrated a weak association, with an *R* ^2^ of 0.12 and a non‐significant coefficient of 0.29 (*p* = 0.14), indicating that 3‐month psychosocial scores did not significantly predict 12‐month outcomes. Similarly, the Spearman correlation yielded a low, non‐significant association (*ρ* = 0.28, *p* = 0.23), suggesting limited temporal stability in psychosocial perceptions over time. In the appearance domain, the 3‐month SCAR‐Q appearance scores were also not significantly associated with 12‐month appearance outcomes. The linear model showed an *R* ^2^ of 0.10 with a coefficient of 0.33 (*p* = 0.18), while the corresponding Spearman correlation was weak and non‐significant (*ρ* = 0.14, *p* = 0.56). These findings suggest that early patient‐perceived aesthetic concerns may fluctuate and may not reliably predict longer‐term scar appearance perceptions.

Collectively, these results underscore the domain‐specific predictive utility of early SCAR‐Q scores. While quality of life, POSAS scores and overall opinion of scar quality are reliably stable and predictive, psychosocial and appearance‐related concerns may evolve over time and thus warrant ongoing assessment and individualised management.

## 4 Discussion

**FIGURE 6:**
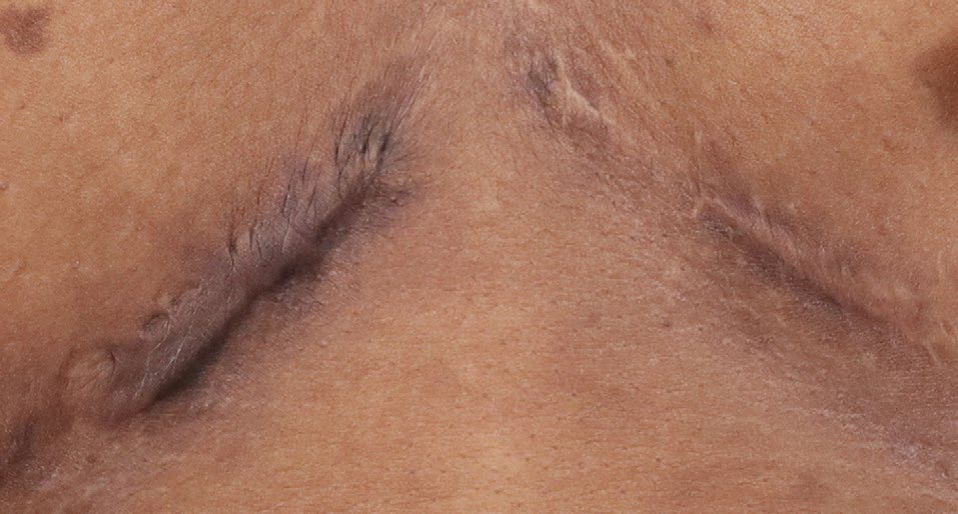
Example of a ‘bad’ scar: patient at 12 months with high Scar‐Q distress scores that worsened on average from 3 months to 12 months. This patient was ‘very bothered’ by how raised the scar looks, ‘very bothered’ by the surface of the scar and ‘very bothered’ by the contour. Her overall rating worsened by 5 points from 4 to 9 in between her 3‐ and 12‐month visits. Her scar was also rated a 7 out of 10 by two surgeons and considered a ‘bad’ scar.

**FIGURE 7:**
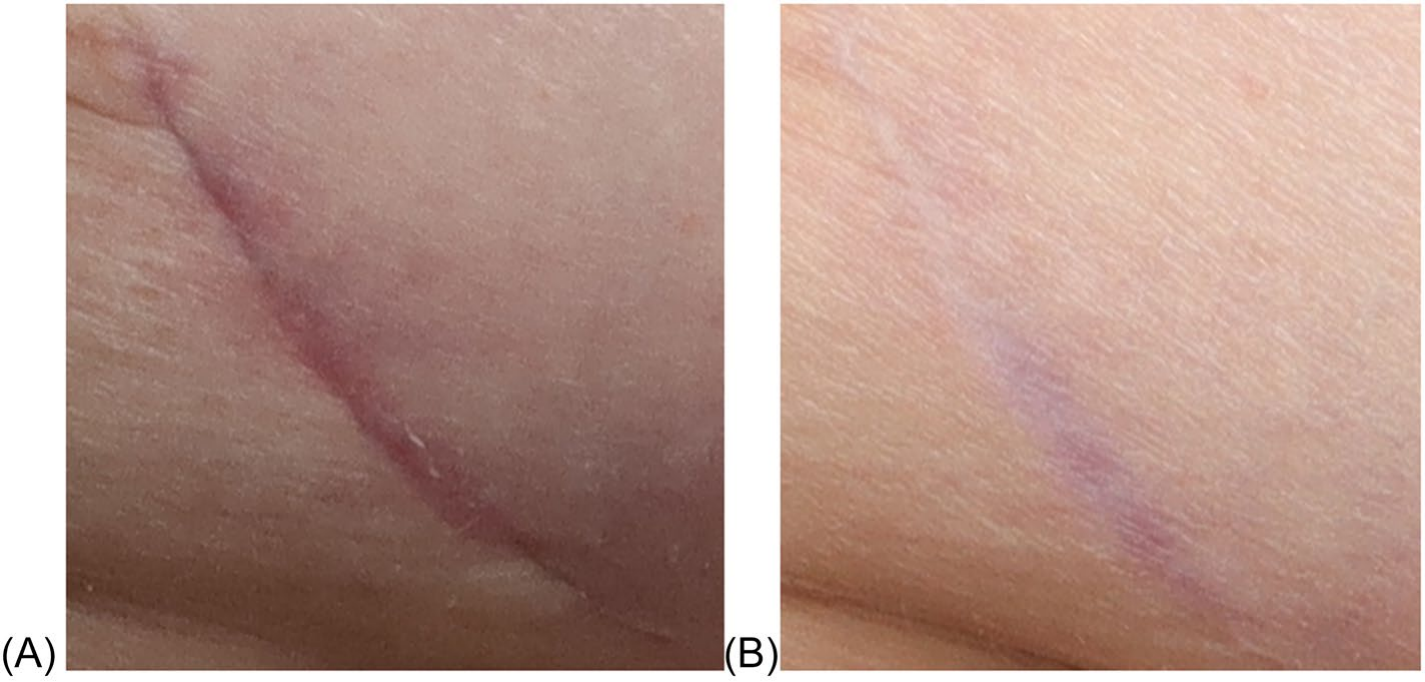
Example of a ‘good’ scar: This patient's scores on the subjective POSAS went down significantly from 10 to 3 for scar colour, 10 to 2 for scar stiffness and scar thickness, 10 to 1 for scar irregularity and good vs. bad scar. At 3 months, she rated her scar a 10 as ‘worst scar imaginable’ but at 12 months, she rated it a 1—normal skin, which is a huge change in PROs across this time difference. However, she was not bothered by the scar itself even though she ranked it so highly for being different from normal skin in many metrics at 3 months.

This study highlights the critical value of early patient‐reported outcome (PRO) measures in understanding and potentially predicting long‐term satisfaction following surgery. As scar evaluation increasingly shifts towards patient‐centred metrics, our findings provide compelling evidence that early PROs—particularly those assessing quality of life (QoL)—can serve as stable, informative indicators of long‐term outcomes. This is especially important given that patient satisfaction is not always aligned with objective scar appearance, and many interventions targeting scar revision or counselling are most effective early in the postoperative period (Figures 6 and 7).

Among the SCAR‐Q domains, QoL emerged as the most temporally stable and predictive. The 3‐month QoL scores explained nearly half of the variance in 12‐month scores (*R* ^2^ = 0.49), with a robust correlation (*ρ* = 0.70, *p* < 0.001), demonstrating that patients' early perceptions of how scars impact their daily lives remain remarkably consistent over time. Although the absolute mean change in QoL was minimal, the relative stability of individual scores implies that early impressions are meaningful and enduring. Clinically, this suggests that assessing QoL as early as 3 months can help identify patients who may continue to struggle with the psychosocial burden of their scars—offering an opportunity for timely psychosocial intervention, support services or education.

In contrast, appearance and psychosocial scores were more variable and less predictive over time, indicating that these domains may be more sensitive to evolving expectations, social contexts or emotional adaptation. Their lower predictive values (*R* ^2^ = 0.10 and 0.12, respectively) and non‐significant correlations suggest that these perceptions are more malleable and may benefit from continued follow‐up, reassurance or treatment adjustments as the scar matures. Importantly, this highlights that not all aspects of patient satisfaction are fixed early in healing—some remain dynamic and open to influence, reinforcing the need for long‐term, multidimensional care strategies.

Importantly, this variability in appearance and psychosocial outcomes underscores the dynamic nature of patient perceptions—what is considered acceptable or bothersome may evolve throughout the healing process. These fluctuations may partly reflect known scar maturation patterns, where parameters such as thickness or stiffness can transiently worsen before improving. As physical characteristics evolve, patients' perceptions of what is considered acceptable or bothersome may evolve throughout the healing process, influenced not only by changes in daily function or emotional adaptation but also by the evolving physical appearance of the scar. This has significant implications for clinical care: rather than assuming a linear trajectory of improved satisfaction with time, clinicians should anticipate potential fluctuations in patient outlook and remain proactive in managing expectations, particularly regarding scar aesthetics and emotional impact. However, the minimal change across all SCAR‐Q scores may reflect the high baseline scores in our cohort and the predominance of small, elective surgical scars, which are less likely to produce marked functional or psychosocial impact.

The correlation analyses further reinforce the need for personalised scar care. Objective scar characteristics at 3 months—especially width and height—demonstrated strong predictive relationships with 12‐month patient‐reported outcomes. For instance, wider scars at 3 months were significantly associated with worse appearance (*ρ* = −0.65, *p* = 0.002) and psychosocial scores (*ρ* = −0.61, *p* = 0.0047) at 12 months. Similarly, greater height at 3 months was linked to poorer aesthetic outcomes and a higher likelihood of ‘bad’ scar classification by patients. These findings suggest that early objective measures can identify patients at risk for persistent dissatisfaction, offering an opportunity for targeted interventions—such as silicone therapy, steroid injections or scar counselling—well before the 12‐month endpoint.

Meanwhile, metrics like depth and vascularity showed minimal associations with long‐term PROs, indicating that not all physical features are equally salient to patients. This reinforces the importance of aligning clinical evaluation with what patients actually value—not merely what is visible or quantifiable.

In our cohort, the POSAS patient scale sum score significantly decreased from 3 to 12 months, indicating overall improvement in patient‐reported scar quality. Item‐level analysis revealed that reductions in stiffness, different colour, and thickness contributed most to this change, aligning with expected scar maturation patterns. These findings suggest that, for many patients, physical improvements in specific scar attributes are reflected in their overall perceptions of quality. Nevertheless, because patient priorities and definitions of an ‘acceptable’ scar can vary, early identification of the attributes most bothersome to each individual remains important for guiding targeted interventions and expectation management throughout the healing process.

Ultimately, these findings support a paradigm shift in postoperative scar care. Rather than relying solely on clinical observation, we advocate for the routine use of patient‐reported outcomes—especially SCAR‐Q QoL and appearance domains—as part of early follow‐up visits. This approach can better identify patients who may need intervention and allows clinicians to tailor care that is both physically restorative and psychosocially supportive. While scars may fade or flatten over time, the patient experience does not always follow the same course. Understanding early perceptions—what bothers patients, how it affects their life, and whether it changes—can inform more empathetic, responsive and effective scar management strategies. This work lays the groundwork for incorporating PROs into surgical outcome prediction models and highlights the value of patient‐centred metrics as both evaluative and prognostic tools in reconstructive care.

### 4.1 Comparison With Existing Literature and Clinical Implications

Our findings align with and extend existing literature underscoring the importance of PROs in scar evaluation. Prior studies, such as those by Mundy et al. and Abelleyra et al., have demonstrated that early PROs—particularly those related to appearance and emotional impact—can forecast long‐term satisfaction [21, 22, 23]. Consistent with this, our results show that early patient perceptions, especially in the quality‐of‐life domain, exhibit strong temporal stability and predictive validity. Additionally, the observed associations between 3‐month scar width and height with 12‐month SCAR‐Q scores reinforce well‐established links between objective scar metrics and subjective distress. However, our study uniquely highlights that not all domains behave uniformly over time—appearance and psychosocial perceptions, for example, may evolve despite objective improvement. This echoes previous work emphasising the psychological complexity of scar healing and the need to contextualise physical outcomes within the patient experience.

Clinically, these results advocate for the routine integration of SCAR‐Q and POSAS in postoperative care. PROs are not only valuable endpoints but also powerful early indicators of future dissatisfaction. Their use enables early identification of at‐risk patients, supports expectation management and promotes tailored interventions. Ultimately, incorporating both physical and psychological measures offers a more comprehensive, patient‐centred approach to scar management [24].

### 4.2 Limitations and Future Directions

This study has several limitations. The small cohort size (*n* = 20) limits generalisability and precludes subgroup analyses. The racial composition was heterogeneous, with most participants identifying as Caucasian and smaller proportions as African‐American or Hispanic, which may have introduced unmeasured confounding given known differences in scar biology and patient perceptions. The within‐subjects longitudinal design, with each participant serving as their own control, enhanced sensitivity to change and reduced inter‐patient variability, allowing adequate statistical power despite the modest cohort. Most scars resulted from elective breast procedures, contributing to high baseline PRO scores and limited variability, further constraining applicability to larger, more complex or traumatic scars. Another limitation is the inclusion of scars from multiple surgical procedures and anatomical sites. While this diversity could affect generalisability, the SCAR‐Q is a validated, site‐agnostic instrument and the within‐subject design helps mitigate procedural heterogeneity. The 3‐month timepoint was chosen to balance early assessment with feasible follow‐up; however, scar pathophysiology begins earlier, and opportunities for preventive intervention may occur before this stage. Scar hypertrophy, inflammation or widening can also develop after 3 months, particularly as patients resume daily activities and mechanical loading at the scar site. These evolving changes may influence both objective measures and patient perceptions, potentially altering the predictive strength of early assessments. Future research should include larger, racially stratified and procedure‐specific cohorts, with extended follow‐up beyond 12 months, to validate these findings and clarify the relationship between early impressions and long‐term scar satisfaction.

## 5 Conclusion

This study underscores the importance of early patient‐reported outcomes in forecasting long‐term perceptions of scar quality. SCAR‐Q quality of life scores at 3 months were particularly robust in predicting 12‐month outcomes, highlighting their utility in tracking stability over time. In contrast, appearance and psychosocial scores showed greater variability, suggesting these domains may be more influenced by evolving expectations, emotional adaptation or external factors. Objective scar metrics demonstrated strong correlations with patient‐reported appearance and psychosocial scores, reinforcing their clinical relevance. The findings support a multidimensional approach to scar assessment that integrates both physical attributes and patient experiences. Incorporating validated PRO instruments like SCAR‐Q and POSAS into postoperative care can facilitate earlier identification of at‐risk patients, guide timely interventions and improve patient satisfaction. Future research should explore longitudinal changes beyond the 12‐month mark and assess the role of psychological resilience, patient education and expectation management in shaping scar‐related outcomes.

## Disclosure

Use of the SCAR‐Q was made under license from McMaster University, Hamilton, Canada.

## Conflicts of Interest

The authors declare no conflicts of interest.

## Data Availability

The data that support the findings of this study are available on request from the corresponding author. The data are not publicly available due to privacy or ethical restrictions.
